# A randomized trial of sodium-restriction on kidney function, fluid volume and adipokines in CKD patients

**DOI:** 10.1186/1471-2369-15-57

**Published:** 2014-04-04

**Authors:** Katrina L Campbell, David W Johnson, Judith D Bauer, Carmel M Hawley, Nicole M Isbel, Michael Stowasser, Jonathan P Whitehead, Goce Dimeski, Emma McMahon

**Affiliations:** 1 Princess Alexandra Hospital, 199 Ipswich Road, Woolloongabba, QLD, Australia; 2 University of Queensland, Brisbane, QLD, Australia; 3 Translational Research Institute, Brisbane, QLD, Australia; 4 Mater Research Institute, The University of Queensland, Brisbane, QLD, Australia; 5 Chemical Pathology, Pathology Queensland, Princess Alexandra Hospital, Brisbane, QLD, Australia

**Keywords:** Dietary sodium, Nutrition, Chronic kidney disease, Cardiovascular disease, Blood pressure, Kidney function, Inflammation

## Abstract

**Background:**

Dietary sodium restriction is a key management strategy in chronic kidney disease (CKD). Recent evidence has demonstrated short-term reduction in blood pressure (BP) and proteinuria with sodium restriction, however the effect on other cardiovascular-related risk factors requires investigation in CKD.

**Methods:**

The LowSALT CKD study involved 20 hypertensive Stage III-IV CKD patients counselled by a dietitian to consume a low-sodium diet (<100 mmol/day). The study was a randomised crossover trial comparing 2 weeks of high-sodium (additional 120 mmol sodium tablets) and low-sodium intake (placebo). Measurements were taken after each crossover arm including BP (peripheral and central), adipokines (inflammation markers and adiponectin), volume markers (extracellular-to-intracellular [E/I] fluid ratio; N-terminal pro-brain natriuretic peptide [NT-proBNP]), kidney function (estimated Glomerular Filtration Rate [eGFR]) and proteinuria (urine protein-creatinine ratio [PCR] and albumin-creatinine ratio [ACR]). Outcomes were compared using paired *t*-test for each cross-over arm.

**Results:**

BP-lowering benefits of a low-sodium intake (peripheral BP (mean ± SD) 148/82 ± 21/12 mmHg) from high-sodium (159/87 ± 15/10 mmHg) intake were reflected in central BP and a reduction in eGFR, PCR, ACR, NTproBNP and E/I ratio. There was no change in inflammatory markers, total or high molecular weight adiponectin.

**Conclusions:**

Short-term benefits of sodium restriction on BP were reflected in significant change in kidney function and fluid volume parameters. Larger, long-term adequately powered trials in CKD are necessary to confirm these results.

**Trial registration:**

Universal Trial Number U1111-1125-2149 registered on 13/10/2011; Australian New Zealand Clinical Trials Registry Number ACTRN12611001097932 registered on 21/10/2011.

## Background

Evidence for the benefits of sodium restriction beyond blood pressure (BP) control has generated interest in the CKD and general population literature. Observational data from non-CKD populations indicates low sodium intake is associated with reduced cardiovascular mortality independent of BP. Intervention studies have also reported benefits of sodium restriction independent of BP, including reduced proteinuria [1,2] and arterial stiffness [3].

Mechanisms driving these changes have yet to be elucidated, however postulated drivers include metabolic changes in systemic inflammation and fluid status [4-7]. Adipokines, including inflammatory makers, have been shown to increase with high sodium diet in the general population [8,9] however this has yet to be tested in kidney disease. Fluid volume derangements occur with sodium loading [10] and may heighten cardiovascular risk through increasing left-ventricular hypertrophy [11]. N-terminal pro-brain natriuretic peptide (NT-proBNP), a biomarker of the cardiac response to volume expansion, is known to be elevated in patients with CKD, and is reduced with a low sodium diet in CKD [12].

Our recent publication from the LowSALT CKD study demonstrated a substantial effect of sodium on increasing BP and proteinuria in Stage III and IV CKD [2]. This current investigation is a post-hoc analysis aiming to assess effects of dietary sodium restriction on kidney function and metabolic markers (kidney function, fluid volume, adipokines).

## Methods

The LowSALT CKD study was a 6-week single-centre double-blind randomized cross-over trial with two 2-week interventions. Methods for this study have been described in detail previously [2,13]. Trial registration numbers for the LowSALT CKD study are: Universal Trial Number U1111-1125-2149, and Australian New Zealand Clinical Trials Registry Number ACTRN12611001097932.

### Participants

Briefly, the target population for the trial was adult hypertensive (BP 130-169/≥70 mmHg), Stage III and IV CKD (GFR 15–59 ml/min/1.73 m^2^) patients attending a single tertiary nephrology service. Exclusion criteria included salt-wasting CKD, pregnant or breastfeeding, current prescription of medications providing >20 mmol sodium per day, life expectancy <6 months, current involvement in another intervention study, or insufficient mental or physical capacity to adhere to the study protocol. Ethical approval was obtained through the Metro South and University of Queensland Human Research Ethics Committee. All participants gave fully informed consent before participation in the study.

### Study design

Participants were counseled by a single, trained dietitian to follow a low sodium diet (aiming for a sodium intake of 60–80 mmol/day) at the commencement of the study. Following a 1-week run-in period, participants were randomized to a high sodium diet (achieved via slow-release sodium tablets providing an additional 120 mmol sodium/day) or low sodium diet (placebo) with a 1-week washout [2,13].

### Outcome assessment

Peripheral (clinic) BP was measured by placing a cuff to the brachial artery in the upper non-dominant arm attached to an oscillometric semiautomatic sphygmomanometer (Omron Healthcare Inc, Illinois, USA) after the patient had been in the seated for at least 5 minutes, taking an average of three consecutive measurements. Central BP was assessed using radial applanation tonometery to acquire an arterial pressure waveform, which was then calculated using SphygmoCorTM CPV software (AtCor Medical, Sydney, Australia) to give a central BP estimation. The operator was blinded to baseline BP results throughout the study.

Peripheral blood was drawn by venopuncture after an overnight fast at each time point. Blood was collected in Greiner Vacuette tubes with gel separator (serum - #456071, lithium heparin plasma - #456083, Kremsmunster, Austria). Blood was centrifuged within 30 minutes after blood collection then serum and plasma were aliquoted into vials and stored at −80°C until analysis. Analysis was performed in batches. Serum creatinine, urate and C-reactive protein (CRP) were measured on Beckman DxC800 general chemistry systems (Beckman Coulter, Brea, CA, USA). In addition, the plasma marker of fluid overload N-Terminal pro-Brain Natriuretic Peptide (NTpro-BNP) was performed on the Roche Elecsys e170 immunoassay system (Roche Diagnostics, Mannheim, Germany).

A range of cytokines were measured. The inflammation markers, interleukin-6, tumor necrosis factor-α and interferon-γ, were measured by an electrochemiluminescence technique using Human Pro-inflammatory Ultra-sensitive Kit with the Sector Imager 6000 (Meso Scale Discovery, Gaithersburg, Maryland, USA). The assay was performed according to the manufacturer’s instructions with CVs of 20% or less considered acceptable, as previously described [14]. Serum concentrations of total and high molecular weight (HMW) and adiponectin were measured in duplicate using ELISA (ALPCO Diagnostics, Salem, NH, USA) according to the manufacturers’ instructions.

Kidney function was estimated using the CKD-EPI formula for estimated Glomerular Filtration Rate (eGFR). 24-hour urine collections were undertaken using standard protocol and analyzed for albumin-creatinine (ACR) and protein-creatinine ratio (PCR) (markers of kidney damage) and total sodium content.

Fluid status was assessed by extracellular-to-intracellular fluid ratio (E/I) and overhydration (in liters) measured using the Body Composition Monitor (BCM) (Fresenius Medical Care, Germany).

Investigators and participants were blinded to outcome measurement throughout the study.

### Statistical analysis

Statistical analyses were performed using STATA (Version 12) with significance set at p < 0.05.

Descriptive data are presented as mean and standard deviation, or median and interquartile range if not normally distributed. Data that were non-normally distributed were transformed prior to analysis. Analysis was undertaken to test paired difference of high versus low sodium intake.

To test for variation due to treatment order, analysis of covariance was conducted with treatment type and treatment order included in the model and observations clustered by study number to account for correlation of within-patient results.

## Results

### Participant characteristics

Participant characteristics and CONSORT diagram were detailed in a previous publication [2]. Briefly, the sample of 20 participants were mean ± SD age 69 ± 11 years, with eGFR 32 ± 12 mL/min per 1.73 m^2^, 75% (n = 15/20) male and 40% with diabetes (n = 9/20).

### Effects of sodium restriction on outcomes

Table 1 demonstrates sodium excretion and BP as well as kidney function, fluid status, and inflammatory markers and adipokines. Peripheral systolic BP was reduced by mean 10 [95% CI 1–20] mm Hg from mean ± SD 159 ± 14 mm Hg at the high sodium period to 148 ± 21 mm Hg at the low sodium period (p = 0.04), while diastolic BP was reduced by 6 [95% CI 1–10] mm Hg from 87 ± 10 mm Hg at the high sodium to 82 ± 12 mm Hg at the low sodium period (p = 0.03). Central systolic BP was reduced by 13 [95% CI 2 – 24] mm Hg from 143 ± 20 mm Hg at the high sodium period to 130 ± 21 mm Hg at the low sodium period (p = 0.03) while central diastolic BP was not significantly reduced (mean change 4 [95% CI −2.2 – 10] mm Hg; from 84 ± 80 mm Hg at the high sodium period to 80 ± 14 mm Hg at the low sodium period; p = 0.20). Central pulse pressure was significantly reduced by 9 [95% CI 2–17] mm Hg from 59 ± 16 mm Hg at the high sodium period to 50 ± 12 mm Hg at the low sodium period (p = 0.02).

**Table 1:** Results from a randomized-crossover trial of sodium restriction in hypertensive CKD patients

	**Baseline**	**High sodium**	**Low sodium**
Sodium excretion (mmol/24 hr)^#^	127 (80–187)	168 (146–219)	75 (58–112)*
**Kidney function**			
eGFR# (mL/min)	32 (23–42)	39 (23–39)	30 (17–36)*
Serum creatinine^#^ (μmol/L)	184 (135–244)	149 (135–230)	172 (157–276)*
Serum urate^#^ (mmol/L)	0.44 (0.40–0.47)	0.39 (0.34–0.45)	0.46 (0.41–0.51)*
Protein: Creatinine (24 h urine)^#^ (g/mol creat)	49 (12–97)	68 (23–164)	41 (17–126)*
Albumin: Creatinine (24 h urine)^#^ (g/mol creat)	21 (2–65)	27 (5–127)	9 (2–82)*
**Volume status**			
Extracellular/Intracellular fluid ratio	0.86 ± 0.14	0.92 ± 0.14	0.87 ± 0.13*
Overhydration (L)^#^	−0.6 (−1.6–1.4)	0.8 (−1.1–2.6)	−0.5 (−1.7–0.9)*
NT–proBNP (pg/mL)^#^	NA	330 (167–793)	205 (124–528)*
**Inflammatory markers**			
C-reactive protein (mg/L)^#^	3.3 (1.6–5.8)	2.8 (1.5–5.5)	2.7 (1.0–7.3)
Interleukin-6 (pg/mL)^#^	NA	1.9 (1.6–2.8)	1.9 (1.4–2.8)
Interferon-gamma (pg/mL)^#^	NA	0.8 (0.5–1.1)	1.0 (0.5–1.4)
Tumor necrosis factor – alpha (pg/mL)^#^	NA	6.8 (5.8–8.7)	7.3 (5.3–9.0)
Total adiponectin (ng/L)	8.1 ± 3.5	7.8 ± 3.6	8.0 ± 3.7
High molecular weight adiponectin (μg/mL)	5.6 ± 3.1	3.9 ± 2.5	3.9 ± 2.6

Data are presented as mean ± standard deviation, median (interquartile range).

NA Data not avaliable.

*p < 0.05 High vs low sodium ^#^log transformed prior to analysis.

Fluid markers were significantly reduced from the high to the low sodium period (p < 0.05). However, inflammatory markers and adipokines were not significantly different between the interventions (p > 0.05) (Table 1). Effect of sodium restriction on outcomes did not vary according to treatment order (p > 0.05).

## Discussion

This study aimed to examine effects of sodium restriction on central and peripheral BP, metabolic markers, markers of kidney function, fluid volume and adipokines. We found that a low sodium dietary intervention resulted in significant reductions in both peripheral and central BP, kidney function and fluid volume, but did not significantly affect inflammatory markers or adiponectin.

Peripheral clinic BP was reduced by mean 10/6 mm Hg from the high salt to the low salt period. This is comparable with ambulatory BP reduction of 10/4 mm Hg as reported in our previous publication [2]. While ambulatory BP is considered to be more strongly related to cardiovascular and renal risk than clinic BP [15,16], reporting both measurements allows for greater comparability with studies using either of these measurements alone.

Measurement of central BP has raised recent interest due to its possibly stronger independent association with cardiovascular events and chronic kidney disease compared with peripheral BP [17]. Safar et al. (2002) measured central BP non-invasively in 180 haemodialysis patients and found that a carotid pulse pressure increase of 25 mm Hg increased the risk of all-cause mortality by 1.4 [95% CI 1.1-1.8] after adjusting for age, dialysis vintage and previous cardiovascular events, while brachial pulse pressure was not a significant predictor of mortality in this model [18]. We found that sodium restriction reduced central systolic BP by 13 mm Hg and central pulse pressure by 9 mm Hg. There is a paucity of data in the pre-end stage CKD population examining clinical outcomes based on this magnitude of change in central BP. However, a recent non-CKD-specific meta-analysis of 6 studies that measured central pulse pressure found that a 10 mmHg increase of central pulse pressure increased the age- and risk-factor-adjusted pooled relative risk for cardiovascular events by 9% (RR 1.09, 95% CI 1.04–1.14) [19]. It is plausible that the reduction in central BP found in this study would be of clinical benefit if maintained long-term. Nevertheless, there is a need for further research specifically in pre-end stage CKD patients showing the effect of long-term central BP changes on cardiovascular remodelling and events.

We also found a significant change in kidney function parameters with a low sodium diet resulting in a decrease in eGFR mirrored by an increase in creatinine and urate, compared with the high sodium diet. This is consistent with findings from other studies investigating the effect of sodium load on creatinine clearance showing that a high sodium intake can result in increased creatinine clearance, at least in the short term [20]. This can be attributed to induced hyperfiltration associated with increased intraglomerular pressure [21,22]. While increasing GFR may be considered a reflection of improved kidney function, inducing a state of hyperfiltration is associated with further kidney damage and longer-term kidney function decline [23,24]. Similarly, protein-to-creatinine and albumin-to-creatinine ratio were both significantly reduced.

Recent controversy has been raised over the effects of a sodium restricted diet on metabolic markers, with findings in non-CKD populations, including recent observational trials in diabetes [25], citing activation of the renin-angiotensin aldosterone system (RAAS) as a potential negative consequence of low sodium intake. Clinical trials have shown that metabolic syndrome modulates the response to dietary sodium intake [26] and that sodium intake could affect plasma adiponectin in healthy men, by modulation of the RAAS [9]. Inhibition of RAAS through ACE-I and ARB therapy results in increased adiponectin, and Liu et al. (2012) recently indicated a high sodium diet resulted in higher adiponectin levels due to suppression of RAAS [27]. Unlike studies in the healhty popualtion [9,28]. In this present study, adipokines were not impacted by sodium intake. There appeared to be no change in inflammatory markers, total or HMW adiponectin. It is possible that our study was underpowered to detect a difference in these markers, in addition to having a short intervention period. There are also the potential confounding factors to account for which are present in CKD, being a chronic inflammatory condition, and resulting in reduced clearance of adipokines [29]. Finally, concurrent treatment with RAAS blockade was prevalant in this cohort with 90% (n = 18/20) of participants in this study were prescribed an ACE-I or alpha-receptor blocker. As it is known that these medications increase adiponectin together with suppression of RAAS [30]. This creates additional challenges in the assessment of change in these markers in this CKD population.

The hypothesis that higher sodium intake has pro-inflammatory activity is physiologically plausible as sodium loading results in tubulointerstitial endothelial inflammation in experimental studies (Dahl rats), independent of haemodynamic changes [31]. Contrary to this, we did not find a significant change in a broad range of cytokines measured during the acute study, despite a significant change in BP and proteinuria [2].

We found a significant change in fluid parameters demonstrating deranged fluid status with a high sodium diet. This volume change was reflected by NT-proBNP increase in response to a high sodium diet. Correspondingly, the high sodium diet resulted in increased extracellular-to-intracellular fluid ratio, increased eGFR and increased urine volume reflecting hyperfiltration and natriuretic response as discussed earlier. The prognostic impact of elevated NT pro-BNP in determining BP and proteinuria response to RAAS blockade (ARB, diuretic and sodium restriction) has been established by Slagman et al. (2012) in proteinuric CKD patients [12]. In this study, NT pro-BNP was reduced by each therapy of the RAAS blockade and dietary sodium restriction. This group identified NT pro-BNP above the reference range of >125 ng/L predicted ‘salt sensitivity’ in terms of both BP and proteinuria response. In our sample, all except one participant had NT pro-BNP levels exceeding 125 and these levels during the low sodium period did not appear to correspond to BP or proteinuria change.

There are a number of limitations to the present study, some of which, including small sample size and short study duration, have previously been discussed [2]. Limitations inherent in non-invasive measurement of central BP were minimized by use of a single operator to reduce observer error, and taking the measurements under controlled circumstances. Some of the discrepancies highlighted between our investigation and others may be due to the range of comorbid factors including moderate-severe CKD present in our population of hypertensive patients. In addition, participants were maintained on their baselne medication regimen throughout the investigation, which may have impacted response to a number of outcomes, including adipokine response. Research with longer follow-up and larger sample size is needed to more confidently elucidate the true effects of sodium restriction on kidney function and metabolic markers.

## Conclusions

The LowSALT CKD study is the first published double-blind randomized controlled trial assessing the effect of salt restriction on BP, central haemodynamics and inflammatory markers in CKD patients. We found that two-weeks of sodium restriction produced statistically and clinically significant reductions in peripheral and central BP, as well as favourable changes in extracellular-to-intracellular volume ratio, reflected by a change in NT-pro-BNP. We found a considerable reduction in eGFR with sodium restriction, potentially attributable to resolution of hyperfiltration, although longer term research is needed to confirm this. Sodium restriction did not significantly change inflammatory markers in the present study, although further research with a larger sample size and longer followup is warranted. The present study adds novel and valuable information regarding the pathogenesis of excessive sodium intake, and benefits of sodium-restriction, in CKD patients.

## Competing interests

The authors declare that they have no competing interests.

## Authors’ contributions

KC conceived the study, designed and conducted the research, analysed the data and drafted the paper; EM conducted the research, analysed the data and drafted the paper; DJ, JD, CH, NI, MS designed the research, assisted with analysis and revised the paper; GD carried out the NT-proBNP assay assisted with analysis and revised the paper; JW led the Adiponectin assay assisted with analysis and revised the paper; KC has primary responsibility for final content; all authors read and approved the final manuscript.

## Pre-publication history

The pre-publication history for this paper can be accessed here:

http://www.biomedcentral.com/1471-2369/15/57/prepub
